# Modeling and classification of gait patterns between anterior cruciate ligament deficient and intact knees based on phase space reconstruction, Euclidean distance and neural networks

**DOI:** 10.1186/s12938-018-0594-1

**Published:** 2018-11-01

**Authors:** Wenbao Wu, Wei Zeng, Limin Ma, Chengzhi Yuan, Yu Zhang

**Affiliations:** 1 Department of Acupuncture, Longyan First Hospital, Longyan, 364000 China; 2 School of Physics and Mechanical & Electrical Engineering, Longyan University, Longyan, 364012 China; 3 Department of Orthopaedic Surgery, Guangzhou General Hospital of Guangzhou Military Command, Guangzhou, 510010 China; 4 Department of Mechanical, Industrial and Systems Engineering, University of Rhode Island, Kingston, RI 02881 USA; 5 Department of Orthopedics, Guangdong General Hospital, Guangdong Academy of Medical Sciences, Guangzhou, 510080 China

**Keywords:** Gait analysis, Anterior cruciate ligament, Movement disorders, Phase space reconstruction (PSR), Euclidean distance (ED), Neural networks

## Abstract

**Background:**

The anterior cruciate ligament (ACL) plays an important role in stabilizing translation and rotation of the tibia relative to the femur. ACL injury alters knee kinematics and usually links to the alternation of gait patterns. The aim of this study is to develop a new method to distinguish between gait patterns of patients with anterior cruciate ligament deficient (ACL-D) knees and healthy controls with ACL-intact (ACL-I) knees based on nonlinear features and neural networks. Therefore ACL injury will be automatically and objectively detected.

**Methods:**

First knee rotation and translation parameters are extracted and phase space reconstruction (PSR) is employed. The properties associated with the gait system dynamics are preserved in the reconstructed phase space. For the purpose of classification of ACL-D and ACL-I knee gait patterns, three-dimensional (3D) PSR together with Euclidean distance computation has been used. These measured parameters show significant difference in gait dynamics between the two groups and have been utilized to form a feature set. Neural networks are then constructed to identify gait dynamics and are utilized as the classifier to distinguish between ACL-D and ACL-I knee gait patterns based on the difference of gait dynamics between the two groups.

**Results:**

Experiments are carried out on a database containing 18 patients with ACL injury and 28 healthy controls to assess the effectiveness of the proposed method. By using the twofold and leave-one-subject-out cross-validation styles, the correct classification rates for ACL-D and ACL-I knees are reported to be 91.3$\%$ and 95.65$\%$, respectively.

**Conclusion:**

Compared with other state-of-the-art methods, the results demonstrate that gait alterations in the presence of ACL deficiency can be detected with superior performance. The proposed method is a potential candidate for the automatic and non-invasive classification between patients with ACL deficiency and healthy subjects.

## Background

Knowledge of spatiotemporal knee motion is important for understanding normal functions as well as addressing clinical problems, including instability after anterior cruciate ligament (ACL) injury. ACL plays an important role in controlling knee joint stability, not only by limiting tibia anterior translation, but also by controlling knee axial rotation and varus movement [1]. Numerous studies have been carried to provide information on biomechanical changes in the ACL-deficient (ACL-D) knees [2–7], which revealed that ACL-D knees would exhibit altered joint kinematics. Currently, the most widely accepted method for assessing joint movement patterns is gait analysis, which offers a unique means of providing insight into mechanisms of ACL-D progression by measuring the kinematic and kinetic parameters [8]. Gait analysis also provides important information concerning motion variability in ACL-D and ACL-intact (ACL-I) knees [9].

Many studies have addressed gait pattern classification and there are several reviews on this subject [10–14]. However, the research work dealing specifically with ACL-D knees is not sufficient [15–18]. Biomechanics plays an important role in the progression of ACL-D knees and many studies have been carried out in gait laboratories to ascertain which parameters are affected by ACL-D knees compared to healthy controls with bilateral ACL-I knees [19–31]. These gait parameters may be adopted as gait features for the classification of gait patterns between ACL-D and ACL-I knees. In the study by Gao et al. [1], spatiotemporal gait and knee joint kinematic variables were calculated and further analyzed. The ACL-D knees exhibited a significant extension deficit compared to the ACL-I knees. A more varus and internally rotated tibial position was also identified in the ACL-D knees during both stair ascent and descent. Knoll et al. [19] revealed a quadriceps-avoidance gait pattern in acute ACL-D patients. Chronic ACL-D individuals demonstrated a significantly different gait pattern. Robinson et al. [32] investigated whether using a direct kinematic or inverse kinematic modeling approach could influence the estimation of knee joint kinematics and kinetics. The similarity between kinematic and kinetic waveforms was evaluated using the root mean square difference and the one-dimensional statistical parametric mapping. Atarod et al. [33] investigated the interactions between different kinematic degree of freedom during normal gait and determined how these interactions would change over time following ACL transection in vivo. They claimed that ACL deficiency would significantly alter the kinematic and kinetic interactions during in vivo gait. Clinical imaging studies of ACL-D individuals versus healthy controls have found greater medial–lateral posterior tibial slope in injured population, with stronger evidence on the lateral plateau slope. To quantify these effects, Marouane et al. [34] used a lower extremity musculoskeletal model which included a detailed finite element model of the knee joint. It was used to compute the role of changes in medial and/or lateral posterior tibial slope on knee joint biomechanics.

The current study has two aims. First, to provide further evidence to support the claim that ACL-D knees demonstrate altered gait patterns compared to ACL-I knees. Second, to provide an automatic and objective method to distinguish between ACL-D and ACL-I knees. Based on the nonlinear and non-stationary nature of knee kinematic signals [35], a popular nonlinear method named phase space reconstruction (PSR), is a valuable tool for the studies of this kind of signals [36–41]. The principle of PSR is to transform the properties of a time series into topological properties of a geometrical object which is embedded in a space, wherein all possible states of the system are represented, each state corresponds to a unique point, and this reconstructed space sharing the same topological properties as the original space. The dynamics in the reconstructed state space is equivalent to the original dynamics. Hence reconstructed phase space is a very useful tool to extract nonlinear dynamics of the signal [36–41]. It is hypothesized that gait dynamics between ACL-D and ACL-I knee gait patterns is significantly different, which implies that PSR offers the potential to compute the difference and classify the two groups.

In this paper, we present a new method using gait analysis to distinguish between ACL-D and ACL-I knees. First knee rotation and translation parameters are extracted and phase space is reconstructed. The properties associated with the gait system dynamics are preserved in the reconstructed phase space. For the purpose of classification of ACL-D and ACL-I knee gait patterns, three-dimensional (3D) PSR together with Euclidean distance (ED) computation has been used. These measured parameters show significant difference in gait dynamics between the two groups and have been utilized to form a feature set. Neural networks are then constructed to identify gait dynamics and are utilized as the classifier, in which the feature set is embedded, to distinguish between ACL-D and ACL-I knee gait patterns based on the difference of gait dynamics between the two groups.

## Methods

**Fig. 1:**
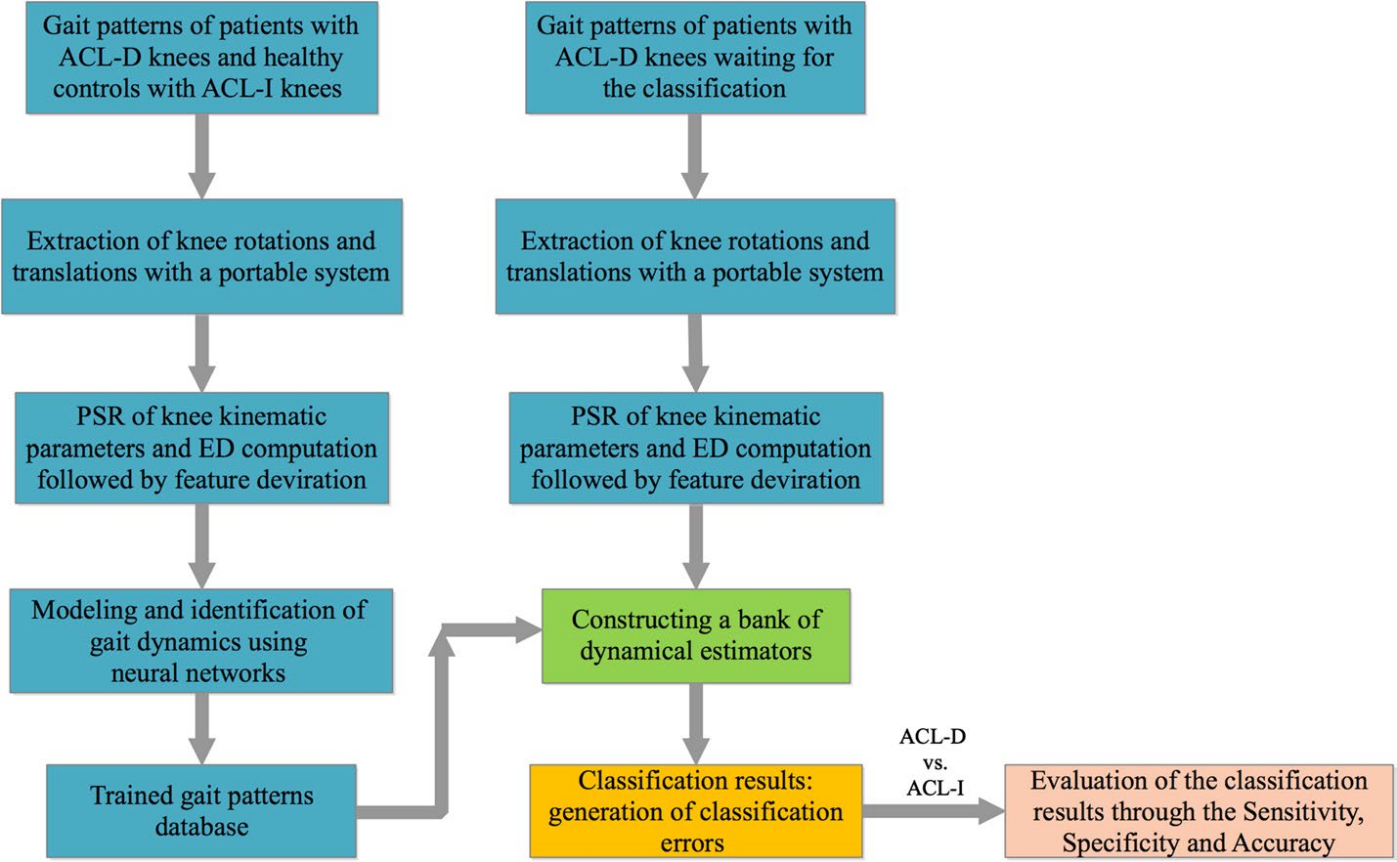
Block diagram of the proposed method for the classification of gait patterns between ACL-D and ACL-I knees

In this section, we propose a method for the classification of ACL-D knees using the information obtained from gait dynamics. Two groups of subjects (patients with ACL-D knees and healthy controls with ACL-I knees) are recruited and tested in this study. The method is divided into the training stage and the classification stage and follows the following steps. In the first step, knee kinematic signals are extracted by using a motion capture system. In the second step, PSR is applied to extract nonlinear dynamics of lower extremities signals. Euclidean distances are computed to extract gait features. Finally, feature vectors are fed into the neural networks for the modeling and identification of gait dynamics. The difference of gait dynamics will be derived from a set of estimators constructed by neural networks and be applied to distinguish between ACL-D and ACL-I knees. The outline of the proposed method is illustrated in Fig. 1.

### Data measurement

Our database consists of 46 participants: 28 healthy controls with ACL-I knees and 18 patients with ACL-D knees. The mean value and the standard deviation (SD) of the age, height, weight and sex for the participants are depicted in Table 1. As the control group, healthy subjects who had bilateral ACL-I knees and no history of musculoskeletal diseases on the lower extremities were included. The ACL-D subjects documented via MRI and a clinical examination had no accompanying damage to the posterior cruciate and collateral ligaments, no more than $30\%$ the meniscus removed, no injuries on the contralateral limb, and no difficulty or pain in performing activities of daily living including walking. A single experienced orthopaedic surgeon performed the physical examination and made the MRI diagnosis.

**Table 1:** Descriptive characteristics of the ACL-D and ACL-I subjects

	Healthy controls with ACL-I knees	Patients with ACL-D knees	*p* value
Age (years), mean (SD)	38.6 (5.9)	40.3 (6.1)	0.352
Height (cm), mean (SD)	165.4 (9.6)	164.1 (7.6)	0.630
Weight (kg), mean (SD)	65.7 (10.5)	63.5 (9.4)	0.474
Male/female	14/14	11/7	−

**Fig. 2:**
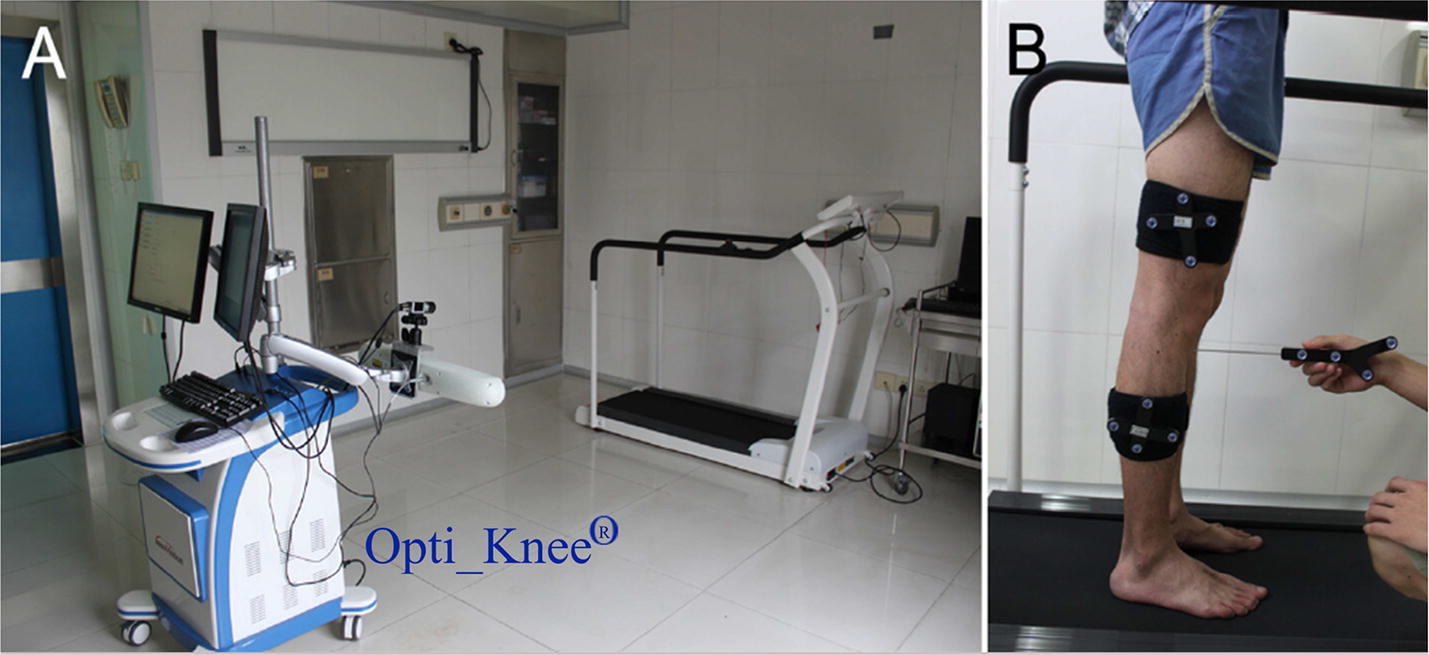
A portable marker-based motion analysis system [42]: **A** The instrument for knee kinematics analysis; **B** Identifying the femoral and tibial anatomical landmarks using a hand-held probe prior to kinematic data capture

The kinematic data of the knees in six-degree-of-freedom (6DOF) were captured using a portable marker-based motion analysis system (Opti_ Knee^®^, Innomotion Inc., Shanghai, China), which has been utilized and validated before [42–45], as illustrated in Fig. 2. These tibiofemoral kinematics include varus–valgus (VV), internal–external (IE) rotation and flexion–extension (FE); anterior–posterior (AP), proximal–distal (PD) and medial–lateral (ML) translations.

Each subject was required to undergo a 3-min treadmill gait training. Then data were collected with the sampling frequency of 60  Hz for 15 s and all the participants were guided to walk at the speed of 3 km/h. The detailed procedure about data extraction can be seen in the study by Zhang et al. [42]. The study was approved by the ethical review board and a written informed consent was obtained from each participant before data collection began.

### Data description

Here in Table 2 we give the measures of the range of motion (ROM) of knee rotations and translations in patients with ACL-D knees and healthy controls with ACL-I knees.

**Table 2:** Mean, SD, significant statistical difference *p* and effect sizes of the range of motion (ROM) of tibiofemoral rotations and translations for 28 healthy controls with ACL-I knees and 18 patients with ACL-D knees

Parameters	Groups		Difference between groups	Effect size
	ACL-D knees	ACL-I knees	*p*-value	Cohen’s *d*
ROM of VV (degree)	13.01 (5.45)	15.40 (4.17)	0.1	0.51
ROM of IE rotation (degree)	18.87 (5.77)	22.45 (4.69)	0.03	0.70
ROM of FE (degree)	59.18 (8.49)	71.76 (6.93)	< 0.001	1.66
ROM of AP translation (cm)	2.41 (0.81)	1.95 (0.52)	0.02	− 0.71
ROM of PD translation (cm)	1.94 (0.74)	2.38 (0.44)	0.01	0.77
ROM of ML translation (cm)	1.84 (0.49)	1.86 (0.37)	0.88	0.05

**Fig. 3:**
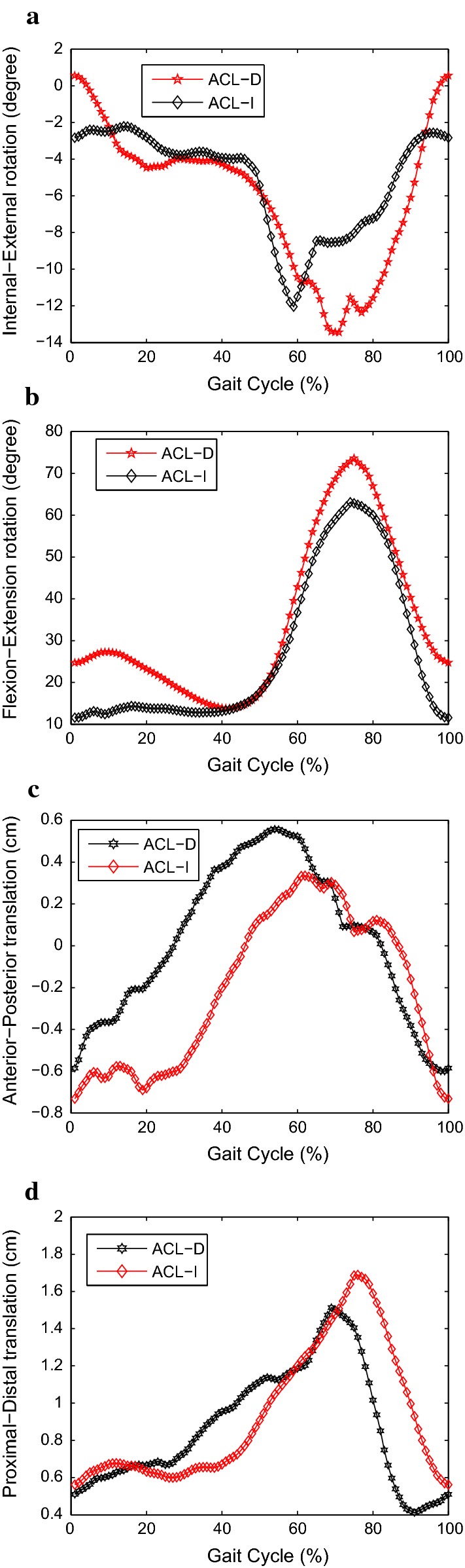
The 3-D joint rotations and translations during walking of ACL-D and ACL-I knees. Ensemble curves of each subject group were normalized from heel strike to heel strike in a gait cycle. **a** IE rotation; **b** FE; **c** AP translation; **d** PD translation

Kinematic variations during walking were observed in 3-D rotations and translations between ACL-D and ACL-I knees, as shown in Fig. 3. For each of the rotational or translational kinematic component, 101 discrete points corresponding to 0–100$\%$ gait cycle at $1\%$ interval were extracted using one-dimensional interpolation for statistical analysis. Measures of each spatiotemporal variable as well as each discrete kinematic point were compared between ACL-D and ACL-I knees using an independent *t*-test analysis of variance (SPSS Inc., IL, USA). A *p* value of $<0.05$ was considered to indicate statistical significance.

It is observed from Table 2 that: (1) In the sagittal plane patients with ACL-D knees showed less range of flexion–extension than healthy controls with ACL-I knees (59.18 (8.49) and 71.76 (6.93), respectively, $p<0.001$). (2) In the frontal plane, patients with ACL-D knees showed less range of internal–external rotation than healthy controls with ACL-I knees (18.87 (5.77) and 22.45 (4.69), respectively, $p=0.03$). (3) The range of PD translation was lower in the ACL-D knees group compared to ACL-I knees group while the range of AP translation was higher in the ACL-D knees group compared to ACL-I knees group (Table 2). (4) Whereas statistical tests of significance tell us the likelihood that experimental results differ from chance expectations, effect-size measurements tell us the relative magnitude of the experimental treatment. In essence, an effect size is the difference between two means divided by the standard deviation of the two conditions [46]. Cohen’s *d* from t-test [47] was used to describe the effect sizes of the ROM of knee kinematic data, which have been shown in Table 2. The effect sizes were traditionally considered small ($d = 0.2$), medium ($d = 0.5$), and large ($d = 0.8$) [48, 49]. It is seen from Table 2 that IE, FE, AP and PD are with nearly large effect sizes compared to VV and ML, which also means there exist significant differences in IE, FE, AP and PD between ACL-deficient patients and healthy controls. The results are in accordance with the *p*-value analysis.

It is seen from the statistical analysis in Table 2 that IE rotation, FE, AP and PD translations between ACL-D and ACL-I knees are significantly different, which means gait dynamics of the two groups represented by the knee motion are significantly different. Hence these four signals are utilized as reference variables to carry out the following phase space reconstruction.

### Phase space reconstruction (PSR)

It is sometimes necessary to search for patterns in a time series and in a higher dimensional transformation of the time series [50]. Phase space reconstruction (PSR) is a method used to reconstruct the so-called phase space. The concept of phase space is a useful tool for characterizing any low-dimensional or high-dimensional dynamic system. A dynamic system can be described using a phase space diagram, which essentially provides a coordinate system where the coordinates are all the variables comprising mathematical formulation of the system. Mathematically, the states of an *d*-dimensional dynamic system can only be characterized by *d* independent quantities. Such a set of *d* independent quantities represents the coordinates of the phase space. One of the most used methods of PSR is the time-delay embedding. Since this method does not require that the treated system could be mathematically defined, explicitly, it fits well with 1-dimensional time series. A point in the phase space represents the state of the system at any given time [50, 51]. Knee kinematic signals can be written as the time series vector $V=\{v_1,v_2,v_3,...,v_K\}$, where *K* is the total number of data points. A new sequence of phase space vectors based on delay-coordinate embedding method is expressed as follows [50]:

1 $$\begin{aligned} Y_j=(V_j,V_{j+\tau },V_{j+2\tau },...,V_{j+(d-1)\tau }) \end{aligned}$$

where $j=1,2,...,K-(d-1)\tau$, *d* is the embedding dimension of the phase space and $\tau$ is a time lag. $Y_j$ means the *j*th reconstructed vector with embedding dimension *d*. Finally, we obtain a reconstructed phase space *Y* containing totally $K-(d-1)\tau$ vector points as the following trajectory matrix:

2 $$\begin{aligned} Y= \left[ \begin{array}{c} Y_1\\ Y_2\\ \cdots \\ Y_M \end{array} \right] = \left[ \begin{array}{cccc} V_1 &{} V_{1+\tau } &{} \cdots &{} V_{1+(d-1)\tau }\\ V_2 &{} V_{2+\tau } &{} \cdots &{} V_{2+(d-1)\tau }\\ \cdots &{} \cdots &{} \cdots &{} \cdots \\ V_M &{} V_{M+\tau } &{} \cdots &{} V_{M+(d-1)\tau } \end{array} \right] \end{aligned}$$

where $M=K-(d-1)\tau$. It is worthwhile to mention that the properties associated with the gait system’s dynamics are preserved in the reconstructed phase space. The *d*-dimensional space of delay coordinates serves as a pseudo state-space which provides a natural setting to approximate the quantitative aspects of the dynamics.

The behavior of the signal over time can be visualized using PSR (especially when $d=$ 2 or 3). In this work, we have confined our discussion to the value of embedding dimension $d=3$, because of their visualization simplicity. For $\tau$ setting, we either utilized the first-zero crossing of the autocorrelation function for each time series or the average $\tau$ value obtained from all the time series in the training dataset by using the method depicted in [52]. In the present study we set the values of time lag $\tau =1$ to test the classification performance. PSR for $d=3$ has been referred as 3D PSR.

3D PSR is the plot of three delayed vectors $V_j,V_{j+1}$ and $V_{j+2}$ to visualize the dynamics of human gait system. Euclidian distance (ED) of a point $(V_j,V_{j+1},V_{j+2})$, which is the distance of the point from origin in 3D PSR and can be defined as [50]

3 $$\begin{aligned} ED_j=\sqrt{V_j^2+V_{j+1}^2+V_{j+2}^2} \end{aligned}$$

ED measures can be used in features extraction and have been studied and applied in many fields, such as clustering algorithms and induced aggregation operators [53].

### Feature extraction and selection

Reconstructed phase spaces have been proven to be topologically equivalent to the original system and therefore are capable of recovering the nonlinear dynamics of the generating system [36, 37]. This implies that the full dynamics of the gait system are accessible in this space, and for this reason, the features extracted from it can potentially contain more and/or different information than the common features extraction method [8]. In order to get a more efficient features set, this paper proposes the following features extraction scheme using ED computation.

1. Reconstruct the phase space for the above mentioned reference variables including knee IE rotation, FE, AP and PD translations with selected values of *d* and $\tau$ for each gait trial;
2. Compute ED of 3D PSR of knee IE rotation, FE, AP and PD translations as gait features. Concatenate these features to form a feature vector $[ED_j^{IE},ED_j^{FE},ED_j^{AP},ED_j^{PD}]^T$ and the dimension of feature space would be four.

**Fig. 4:**
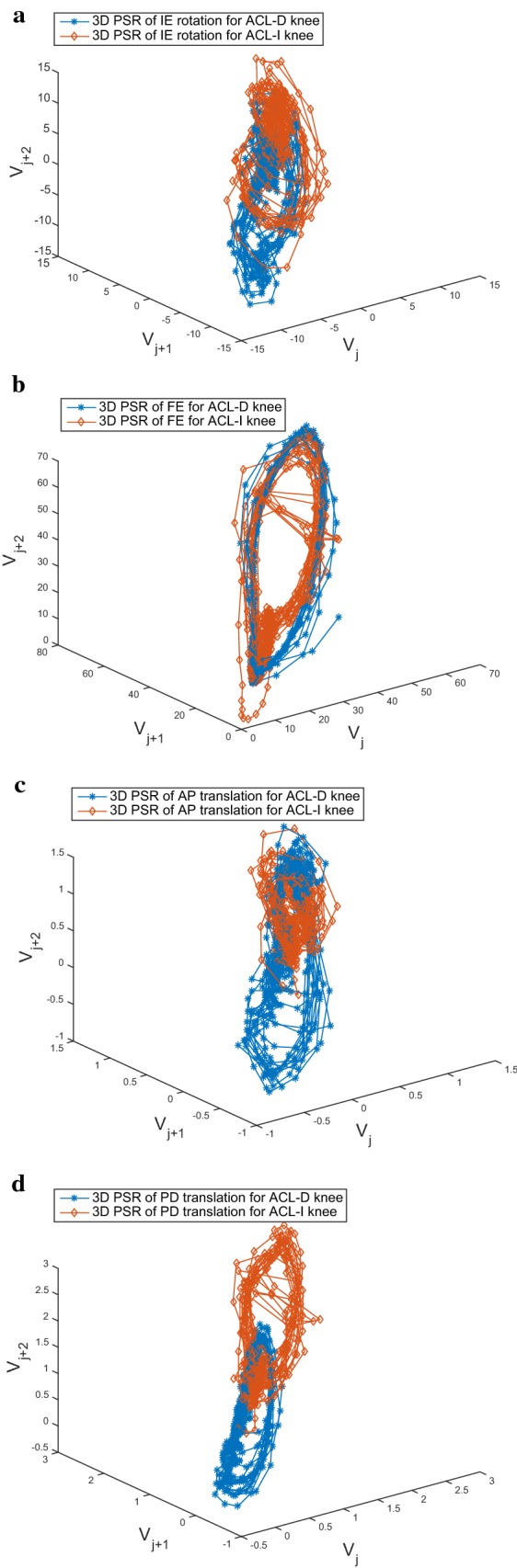
Samples of 3D PSR of the knee kinematic signals from ACL-D and ACL-I gait patterns: **a** 3D PSR of the IE rotation; **b** 3D PSR of the FE; **c** 3D PSR of the AP translation; **d** 3D PSR of the PD translation

**Fig. 5:**
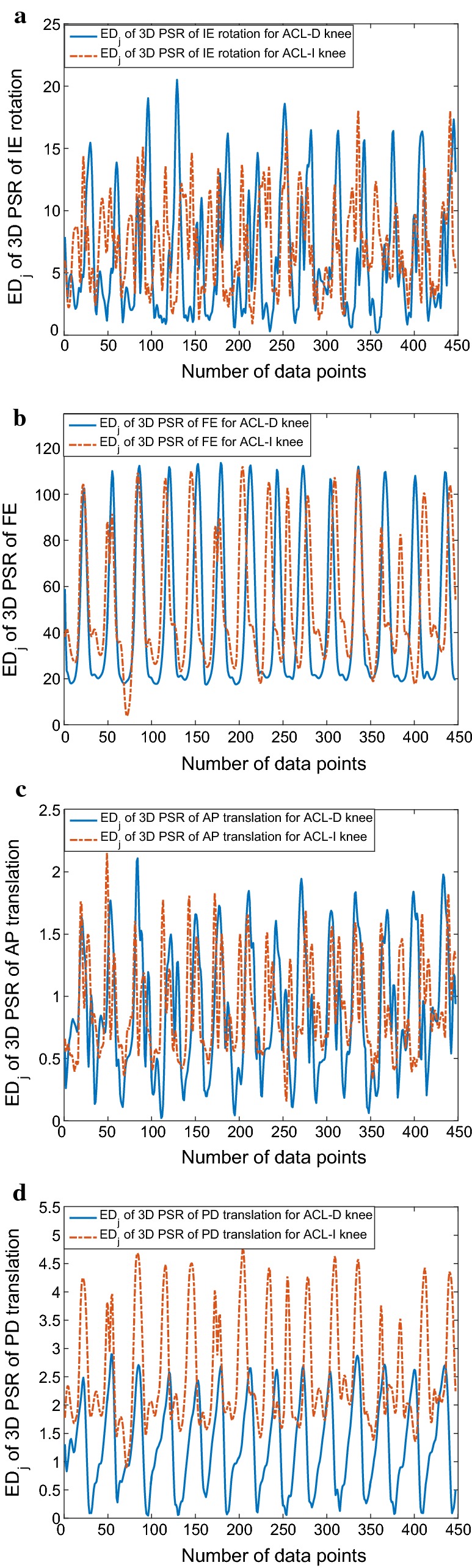
Samples of Euclidian distance of 3D PSR of the knee kinematic signals from ACL-D and ACL-I gait patterns: **a** Euclidian distance of 3D PSR of the IE rotation; **b** Euclidian distance of 3D PSR of the FE; **c** Euclidian distance of 3D PSR of the AP translation; **d** Euclidian distance of 3D PSR of the PD translation

For our dataset, IE rotation, FE, AP and PD translations of two groups (ACL-D and ACLI knees) are analyzed and signal dynamics are extracted by using 3D PSR. Samples of the 3D PSR of knee IE rotation, FE, AP and PD translations are exhibited in Fig. 4. After 3D PSR, features of $[ED_j^{IE},ED_j^{FE},ED_j^{AP},ED_j^{PD}]^T$ for ACL-D and ACL-I knee gait patterns are derived through ED computation, as shown in Fig. 5. As we have analyzed before, significant difference in knee gait dynamics have been reported between ACL-D and ACL-I knees, which can also be seen obviously from Fig. 4.

### Training and modeling mechanism based on selected features

In this section, we present a scheme for modeling and identification of gait dynamics of ACL-I and ACL-D knees based on the above mentioned features.

Consider a general nonlinear human gait system dynamics in the following form:

4 $$\begin{aligned} \dot{x}=F(x;p)+v(x;p) \end{aligned}$$

where $x=[x_1,\ldots ,x_n]^T\in R^n$ are the system states which represent the features $[ED_j^{IE},ED_j^{FE},ED_j^{AP},ED_j^{PD}]^T$, *p* is a constant vector of system parameters. $F(x;p)=[f_1(x;p),\ldots ,f_n(x;p)]^T$ is a smooth but unknown nonlinear vector representing the gait system dynamics, *v*(*x*; *p*) is the modeling uncertainty. Since the modeling uncertainty *v*(*x*; *p*) and the gait system dynamics *F*(*x*; *p*) cannot be decoupled from each other, we consider the two terms together as an undivided term, and define $\phi (x;p):=F(x;p)+v(x;p)$ as the general gait system dynamics. Then, the following steps are taken to model and derive the gait system dynamics via deterministic learning theory [54–56].

In the first step, standard RBF neural networks are constructed in the following form

5 $$\begin{aligned} f_{nn}(Z)=\sum \limits _{i=1}^N w_is_i(Z)=W^TS(Z), \end{aligned}$$

where *Z* is the input vector, $W=[w_1,...,w_N]^T\in R^N$ is the weight vector, *N* is the node number of the neural networks, and $S(Z)=[s_1(\parallel Z-\mu _1\parallel ),..., s_N(\parallel Z-\mu _N\parallel )]^T$, with $s_i(\parallel Z-\mu _i\parallel )=\exp [\frac{-(Z-\mu _i)^T(Z-\mu _i)}{\eta _i^2}]$ being a Gaussian function, $\mu _i(i=1,...,N)$ being distinct points in state space, and $\eta _i$ being the width of the receptive field.

In the second step, the following dynamical RBF neural networks are employed to model and derive the gait system dynamics $\phi (x;p)$:

6 $$\begin{aligned} \dot{\hat{x}}=-A(\hat{x}-x)+\hat{W}^TS(x) \end{aligned}$$

where $\hat{x}=[\hat{x}_1,\ldots ,\hat{x}_n]$ is the state vector of the dynamical RBF neural networks, $A=diag[a_1,\ldots ,a_n]$ is a diagonal matrix, with $a_i>0$ being design constants, localized RBF neural networks $\hat{W}^TS(x)=[\hat{W}_1^TS_1(x),\ldots ,\hat{W}_n^TS_n(x)]^T$ are used to approximate the unknown $\phi (x;p)$.

The following law is used to update the neural weights

7 $$\begin{aligned} \dot{\hat{W}}_i=\dot{\tilde{W}}_i=-\Gamma _iS(x)\tilde{x}_i-\sigma _i\Gamma _i\hat{W}_i \end{aligned}$$

where $\tilde{x}_i=\hat{x}_i-x_i, \tilde{W}_i=\hat{W}_i-W_i^*$, $W_i^*$ is the ideal constant weight vector such that $\phi _i(x;p)={W_i^*}^TS(x)+\epsilon _i(x)$, $\epsilon _i(x)<\epsilon ^*$ represents the neural network modeling error, $\Gamma _i=\Gamma _i^T>0$, and $\sigma _i>0$ is a small value.

With Eqs. (4−6), the derivative of the state estimation error $\tilde{x}_i$ satisfies

8 $$\begin{aligned} \dot{\tilde{x}}_i=-a_i\tilde{x}_i+\hat{W}_i^TS(x)-\phi _i(x;p)=-a_i\tilde{x}_i+\tilde{W}_i^TS(x)-\epsilon _i \end{aligned}$$

In the third step, by using the local approximation property of RBF neural networks, the overall system consisting of dynamical model (8) and the neural weight updating law (7) can be summarized into the following form in the region $\Omega _\zeta$

9 $$\begin{aligned} \left[ \begin{array}{c} \dot{\tilde{x}}_i\\ \dot{\tilde{W}}_{\zeta i} \end{array} \right] = \left[ \begin{array}{cc} -a_i&{}S_{\zeta i}(x)^T\\ -\Gamma _{\zeta i}S_{\zeta i}(x)&{}0 \end{array} \right] \left[ \begin{array}{c} \tilde{x}_i\\ \tilde{W}_{\zeta i} \end{array} \right] + \left[ \begin{array}{c} -\epsilon _{\zeta i}\\ -\sigma _i\Gamma _{\zeta i}\hat{W}_{\zeta i} \end{array} \right] \end{aligned}$$

and

10 $$\begin{aligned} \dot{\hat{W}}_{\bar{\zeta }i}=\dot{\tilde{W}}_{\bar{\zeta }i}=-\Gamma _{\bar{\zeta }i}S_{\bar{\zeta }i}(x)\tilde{x}_i-\sigma _i\Gamma _{\bar{\zeta }i}\hat{W}_{\bar{\zeta }i} \end{aligned}$$

where $\epsilon _{\zeta i}=\epsilon _i-\tilde{W}_{\bar{\zeta }i}^TS_{\bar{\zeta }}(x)$. The subscripts $(\cdot )_\zeta$ and $(\cdot )_{\bar{\zeta }}$ are used to stand for terms related to the regions close to and far away from the trajectory $\varphi _\zeta (x_0)$. The region close to the trajectory is defined as $\Omega _\zeta :={Z|\mathrm {dist}(Z,\varphi _\zeta )\le d_{\iota }}$, where $Z=x, d_\iota >0$ is a constant satisfying $s(d_\iota )>\iota$, $s(\cdot )$ is the RBF used in the network, $\iota$ is a small positive constant. The related subvectors are given as: $S_\zeta (x)=[s_{j1}(x),\ldots ,s_{j\zeta }(x)]^T\in R^{N_\zeta }$, with the neurons centered in the local region $\Omega _\zeta$, and $W_\zeta ^*=[w_{j1}^*,\ldots ,w_{j\zeta }^*]^T\in R^{N_\zeta }$ is the corresponding weight subvector, with $N_\zeta <N$. For localized RBF neural networks, $|\tilde{W}_{\bar{\zeta }i}^TS_{\bar{\zeta }}(x)|$ is small, so $\epsilon _{\zeta i}=O(\epsilon _i)$.

Finally, according to Theorem 1 in [57], the regression subvector $S_{\zeta i}(x)$ satisfies the persistent excitation condition almost always. This will lead to exponential stability of $(\tilde{x}_i,\tilde{W}_{\zeta i})=0$ of the nominal part of system (9) [58]. Based on the analysis results given in [57], the neural network weight estimate error $\tilde{W}_{\zeta i}$ converges to small neighborhoods of zero, with the sizes of the neighborhoods being determined by $\epsilon _{\zeta i}$ and $\Vert \sigma _i\Gamma _{\zeta i}W_{\zeta i}^*\Vert$, both of which are small values. This means that the entire RBF network $\hat{W}_i^TS(x)$ can approximate the unknown $\phi _i(x;p)$ along the trajectory $\varphi _\zeta$, and

11 $$\begin{aligned} \phi _i(x;p)=\hat{W}_i^TS(x)+\epsilon _{i1} \end{aligned}$$

where $$\epsilon _{i1}=O(\epsilon _{\zeta i})$$.

By the convergence result, we can obtain a constant vector of neural weights according to

12 $$\begin{aligned} \bar{W}_i=mean_{t\in [t_a,t_b]}\hat{W}_i(t) \end{aligned}$$

where $t_b>t_a>0$ represent a time segment after the transient process. Therefore, we conclude that accurate identification of the function $\phi _i(x;p)$ is obtained along the trajectory $\varphi _\zeta (x_0)$ by using $\bar{W}_i^TS_i(x)$, i.e.,

13 $$\begin{aligned} \phi _i(x;p)=\bar{W}_i^TS(x)+\epsilon _{i2} \end{aligned}$$

where $\epsilon _{i2}=O(\epsilon _{i1})$ and subsequently $\epsilon _{i2}=O(\epsilon ^*)$.

### Classification mechanism

In this section, we present a scheme to distinguish between ACL-I and ACL-D knees.

Consider a training dataset consisting of gait patterns $\varphi _\zeta ^k$, $k=1,\ldots ,M$, with the *kth* training pattern $\varphi _\zeta ^k$ generated from

14 $$\begin{aligned} \dot{x}=F^k(x;p^k)+v^k(x;p^k),~~x(t_0)=x_{\zeta 0} \end{aligned}$$

where $F^k(x;p^k)$ denotes the gait system dynamics, $v^k(x;p^k)$ denotes the modeling uncertainty, $p^k$ is the system parameter vector.

As shown in the above subsection, the general gait system dynamics $\phi ^k(x;p^k):=F^k(x;p^k)+v^k(x;p^k)$ can be accurately derived and preserved in constant RBF neural networks $\bar{W}^{k^T}S(x)$. By utilizing the learned knowledge obtained in the training stage, a bank of *M* estimators is constructed for the training gait patterns as follows:

15 $$\begin{aligned} \dot{\bar{\chi }}^k=-B(\bar{\chi }^k-x)+\bar{W}^{k^T}S(x) \end{aligned}$$

where $k=1,\ldots ,M$ is used to stand for the *kth* estimator, $\bar{\chi }^k=[\bar{\chi }_1^k,\ldots ,\bar{\chi }_n^k]^T$ is the state of the estimator, $B=diag[b_1, \ldots , b_n]$ is a diagonal matrix which is kept the same for all estimators, *x* is the state of an input test gait pattern generated from Eq. (4).

In the classification phase, by comparing the test gait pattern (standing for an ACL-D or an ACL-I gait pattern) generated from gait system (4) with the set of *M* estimators (15), we obtain the following test error systems:

16 $$\begin{aligned}&\dot{\tilde{\chi }}_i^k=-b_i\tilde{\chi }_i^k+\bar{W}_i^{k^T}S_i(x)-\phi _i(x;p),\nonumber \\&\quad i=1,\ldots ,n,~~k=1,\ldots ,M \end{aligned}$$

where $\tilde{\chi }_i^k=\bar{\chi }_i^k-x_i$ is the state estimation (or synchronization) error. We compute the average $L_1$ norm of the error $\tilde{\chi }_i^k(t)$

17 $$\begin{aligned} \Vert \tilde{\chi }_i^k(t)\Vert _1=\frac{1}{\mathrm {T}_c}\int _{t-\mathrm {T}_c}^t|\tilde{\chi }_i^k(\tau )|d\tau ,~~~t\ge \mathrm {T}_c \end{aligned}$$

where $\mathrm {T}_c$ is the cycle of human gait.

The fundamental idea of the classification between ACL-D and ACL-I knees is that if a test gait pattern generated from a certain ACL-D or ACL-I knee is similar to the trained gait pattern $s~(s\in \{1,\ldots ,k\})$, the constant RBF network $\bar{W}_i^{s^T}S_i(x)$ embedded in the matched estimator *s* will quickly recall the learned knowledge by providing accurate approximation to gait system dynamics. Thus, the corresponding error $\Vert \tilde{\chi }_i^s(t)\Vert _1$ will become the smallest among all the errors $\Vert \tilde{\chi }_i^k(t)\Vert _1$. Based on the smallest error principle, the appearing test gait pattern can be classified. We have the following classification scheme.

**Classification scheme**: If there exists some finite time $t^s,~s\in \{1,\ldots ,k\}$ and some $i\in \{1,\ldots ,n\}$ such that $\Vert \tilde{\chi }_i^s(t)\Vert _1<\Vert \tilde{\chi }_i^k(t)\Vert _1$ for all $t>t^s$, then the appearing gait pattern can be classified.

## Experimental results

The classification performance of ACL-D knees against ACL-I knees is evaluated on several experiments. Three measurements, including the Sensitivity, the Specificity and the Accuracy, are employed for the evaluation, which are defined as follows:

18 $$\begin{aligned}&\mathrm{Sensitivity}=\frac{TP}{TP+FN} \end{aligned}$$

19 $$\begin{aligned}&\mathrm{Specificity}=\frac{TN}{TN+FP} \end{aligned}$$

20 $$\begin{aligned}&\mathrm{Accuracy}=\frac{TP+TN}{TP+TN+FN+FP} \end{aligned}$$

where TP is the number of true positives, FN is the number of false negatives, TN is the number of true negatives and FP is the number of false positives.

The classification results of ACL-D knees will be evaluated in the twofold cross-validation and leave-one-subject-out cross-validation styles, respectively. In the experiment of twofold cross-validation style, we randomly select half of the group of patients with ACL-D knees and half of the group of the healthy controls with ACL-I knees to constitute the training dataset, the rest of the subjects in the two groups are selected as the test dataset. That means there are 9 patients with ACL-D knees and 14 healthy controls with ACL-I knees in the training dataset. In the experiment of leave-one-subject-out cross-validation, each time we select one subject for classification, the rest of the 45 subjects for training. This process is repeated 46 times and the leave-one-subject-out classification accuracy is calculated as the average of the classification accuracy of all of the individually left-out subjects.

In the training phase, the RBF network $\hat{W}_i^TS_i(x)$ is constructed in a regular lattice, with nodes $N=83521$, the centers $\mu _i$ evenly spaced on $[-1.2,1.2]\times [-1.2,1.2]\times [-1.2,1.2]\times [-1.2,1.2]$ so as to cover all the trajectories of the input vectors, and the widths $\eta =0.15$. The weights of the RBF neural networks are updated according to Eq. (7). The initial weights $\hat{W}_i(0)=0$. The design parameters for (6) and (7) are $a_i=0.5, \Gamma =diag\{1.5,1.5,1.5,1.5\}, \sigma _i=10, (i=1,\ldots ,4)$.

**Fig. 6:**
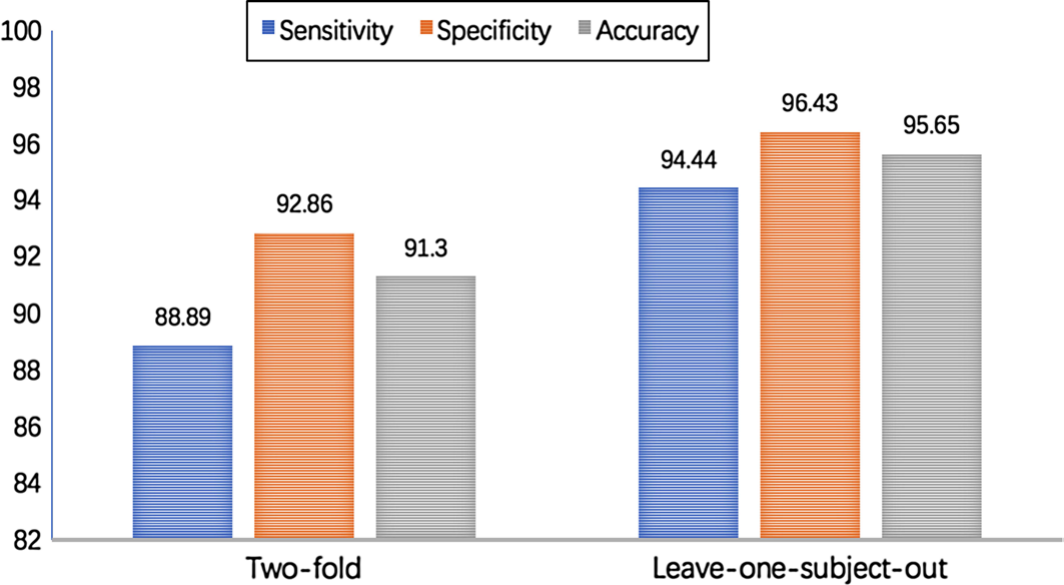
Performance of the proposed classification approach evaluated by the twofold cross-validation and leave-one-subject-out cross-validation methods

In the classification phase, by using the constant networks $\bar{W}_i^{k^T}S_i(x)$, RBF network estimators are constructed based on Eq. (15). The parameters in Eqs. (15) and (17) are $b_i=-30~(i=1,\ldots ,4), T_c=1.08s$. Experimental results are illustrated in Tables 3 and 4, and Fig. 6. Tables 3 and 4 shows the confusion matrix of gait pattern classification between ACL-D and ACL-I knees by using twofold and leave-one-subject-out cross-validation styles. Figure 6 shows the classification results. By using the twofold cross-validation and leave-one-subject-out cross-validation styles, the correct classification rates for ACL-D knees are reported to be 91.3$\%$ and 95.65$\%$, respectively.

**Table 3:** Confusion matrix of gait pattern classification between ACL-D and ACL-I knees by using twofold cross-validation method

	ACL-D knees	ACL-I knees
ACL-D knees	8	1
ACL-I knees	1	13

**Table 4:** Confusion matrix of gait pattern classification between ACL-D and ACL-I knees by using leave-one-subject-out cross-validation method

	ACL-D knees	ACL-I knees
ACL-D knees	17	1
ACL-I knees	1	27

## Discussion

**Fig. 7:**
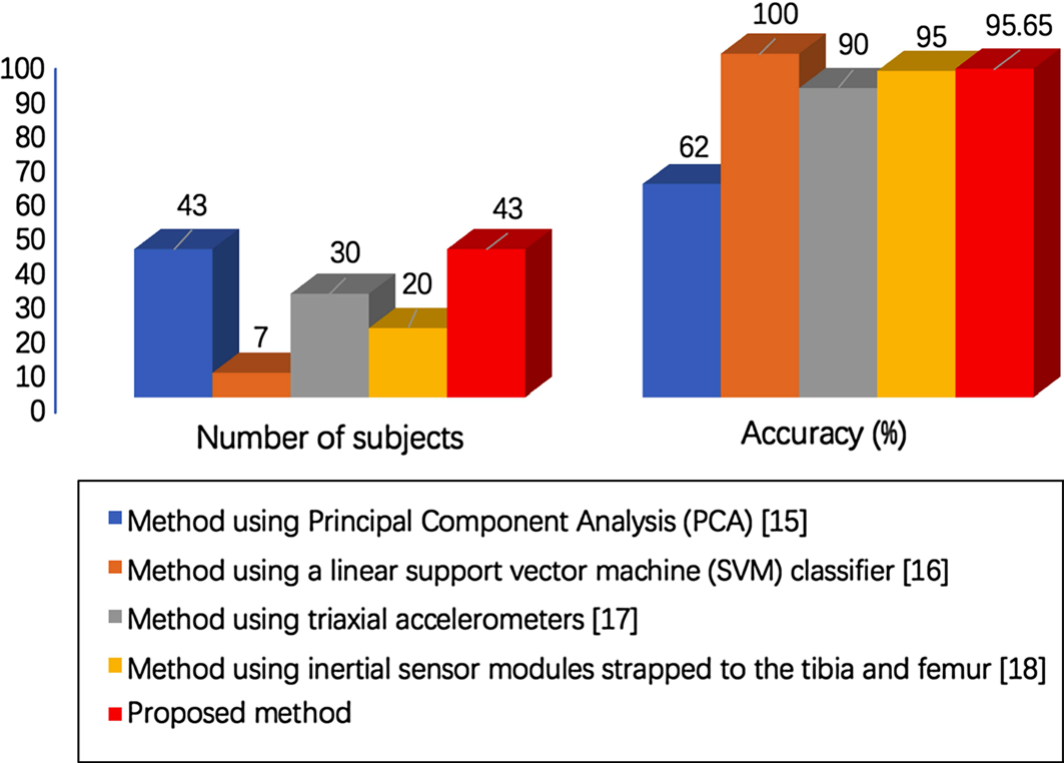
Comparing the results of accuracy in classifying gait patterns between ACL-I and ACL-D groups using different methods

The methodology described in this study is expected to provide the clinicians with an efficient tool for assisted diagnosis of ACL-D knees. In comparison to other methods reported in [15, 16, 20, 24, 59–61], the proposed method focuses not only on providing evidence to support the claim that ACL-D knees demonstrate altered gait patterns compared to ACL-I knees, but also on providing an automatic and objective method to distinguish between patients with ACL-D knees and healthy controls with ACL-I knees. Almosnino et al. [15] aimed to identify, using Principal Component Analysis, strength curve features that explain the majority of variation between the injured and uninjured knee, and to assess the capabilities of these features to detect the presence of injury. 43 unilateral ACL deficient patients were included in the experiments to discern between the ACL-D and contra lateral, healthy knees. The specificity, sensitivity and accuracy are reported to be 60.5$\%$, 60.5$\%$ and 62$\%$, respectively. Christian et al. [16] showed the potential of a pattern recognition system for the diagnoses of kinematic gait patterns in patients due to a recently ruptured ACL. Principal component analysis and recursive feature elimination were used to extract features from 3D marker trajectories. Seven patients with acute ACL rupture were included in the experiment and cross validation yielded $100\%$ accuracy. However, the database used is too small which may weaken the persuasion of the classification performance. Berruto et al. [17] used tibial accelerometers to quantify pivot-shift differences between knees for subjects with unilateral ACL injuries. They considered only acceleration-based metrics, and discrimination of the side of ACL deficiency was accomplished by comparing the magnitudes of accelerations measured for the two legs. Accuracy of correctly identifying the injured knee was roughly $90\%$. Kopf et al. [18] performed a study similar to [17], in which 20 subjects with unilateral ACL deficiency were graded with inertial sensor modules strapped to the tibia and femur. All 3 metrics based on accelerometer measurements were found to be significantly different between injured and uninjured knees of subjects with unilateral ACL deficiency. They did not explicitly state accuracies in determining the side of ACL injury, but examination of their results suggested an accuracy of 95$\%$ (19 of 20) based on acceleration difference. Comparison of the classification performance to other state-of-the-art methods between ACL-I and ACL-D groups is shown in Fig. 7.

Different from the methods in the above-mentioned literature, our method focused on modeling the human gait and extracting the disparity of gait system dynamics between ACL-D and ACL-I knees for the discrimination task. It abandoned the traditional and direct comparison of lower extremity motion parameters between ACL-D and ACL-I knees and adopted instead the modeling, identification and classification of gait dynamics based on motion parameters. This may better explain and reveal the motion principle of pathological and healthy gaits hidden underneath the parameters extracted through PSR and ED. The proposed method serves not only as a measure of kinematic variability and discrimination between two groups of patients with ACL deficiency and healthy controls, but also as a non-invasive, objective and assistant technical means to other diagnostic approaches such as X-rays, MRI, arthroscopy, etc.

However, there are some limitations in the present study which need further improvement. Experiments were carried out on a small database and more participants need to be recruited to verify the effectiveness. At current stage, the proposed method is more suitable to be a tool applicable for gait reeducation on previously diagnosed patients. It is not easy for the clinicians to distinguish a deficiency of the ACL from a possible injury of another structure of the knee, such as posterior cruciate ligament or collateral ligaments, since these injuries may also lead to the same gait patterns. Only when the patients were highly suspected to have the ACL injury, can the proposed method be used to diagnose it as an assistant tool. In future work, injury of other structures of the knee, including posterior cruciate ligament or collateral ligaments injury and their related gait patterns, may also be included in our study to assist in diagnosing the knee injury more accurately. In addition, other parameters regarding different knee lessons can be adopted to improve the classification accuracy.

## Conclusions

The results of this study indicate that the pattern classification of knee kinematic data can offer an objective and invasive method to assess the gait disparity between ACL-D and ACL-I knees. These results demonstrate the potential of the proposed technique for detecting pathological gait patterns caused by ACL deficiency by analysing and measuring the disparity of gait system dynamics using PSR, ED and neural networks. PSR is one of the most used methods which is the time-delay embedding and fits well with 1-dimensional time series. The *d*-dimensional space of delay coordinates serves as a pseudo state-space which provides a natural setting to approximate the quantitative aspects of the gait system dynamics. PSR plots gait system dynamics along the gait signal trajectory in a 3D phase space diagram and visualizes the gait system dynamics. ED measures and derives gait features, which are fed into RBF neural networks for the modeling, identification and classification of gait system dynamics between ACL-D and ACL-I knees. However, some limitations such as the small size of the database, the regulation principle of the embedding dimension and time lag, still need to be improved and overcome. Future work will include a clinical validation of the proposed technique with a larger number of patients with ACL deficiency and age-matched healthy controls. In the present study, PSR parameters such as the time lag and embedding dimension are with fixed values. Assessments of the relationship between the embedding dimension, time lag and the classification accuracy can also be considered in future investigations.
